# The complete mitochondrial genome of Korean endemic loach *Iksookimia pacifica* (Teleostei, Cypriniformes, Cobitidae)

**DOI:** 10.1080/23802359.2022.2093677

**Published:** 2022-07-08

**Authors:** Seung Woon Yun, Hyun Tae Kim

**Affiliations:** a Faculty of Biological Science, College of Natural Science, Jeonbuk National University, Jeonju, South Korea; b Department of Science Education, Jeonju National University of Education, Jeonju, South Korea

**Keywords:** *Iksookimia pacifica*, complete mitochondrial genome, phylogeny

## Abstract

The complete mitochondrial genome of a Korean endemic species, *Iksookimia pacifica* (Teleostei: Cypriniformes: Cobitidae) was sequenced using the NGS method. Its total mitogenome was 16,561 bp in length, comprising 13 protein-coding genes (PCGs), 2 ribosomal RNA genes (rRNA), 22 transfer RNA genes (tRNA), and 1 control region (D-loop). The gene order and content were congruent with those of other cobitid species. In the phylogenetic tree using the maximum likelihood method, *I. pacifica* was clearly distinguished and most closely related to *I. koreensis*. The mitogenome sequence data of *I. pacifica* will provide useful information on the phylogenetic relationship among Cobitidae species.

*Iksookimia pacifica* was previously reported as a synonym for *Cobitis tenia granoei* (Kim 1980) and *C. melanoleuca* (Nalbant 1993), but it was newly classified as *C. pacifica* based on its body pigmentation and morphological characteristics (Kim et al. 1999). However, recently it was reclassified into genus *Iksookimia* through a taxonomic review of the family Cobitidae by Kim (2009). *Iksookimia pacifica* is an endemic species and inhabits only the eastern part of the Korean Peninsula, so analyzing their genetic information is very important for conservation biology. Therefore, here we reported the first complete mitochondrial genome of *I. pacifica* obtained from the fin clip and analyzed its phylogenetic relationship with the other cobitid species.

Specimens of *I. pacifica* were collected in Goseong, South Korea (38°18′29.35″N, 128°31'40.82″E) in 2021. The specimen was deposited at Environmental Biology Laboratory, Jeonbuk National University in Korea under the voucher number JBNU 39128 (Hyun Tae Kim, ecoscience@jbnu.ac.kr). For genomic DNA analysis, a piece of pectoral fin was dissected from the specimen and stored in 100% ethyl alcohol. After that, the total DNA was purified with the genomic DNA Prep Kit for blood and tissue (QUIAGEN Co., USA). Sequencing was performed using the Illumina Hi-Seq X-10 platform (San Diego, CA, USA), and the *de novo* assembler SPAdes 3.13.0 (Bankevich et al. 2012) was conducted for mitogenome construction. The mitogenome sequence was annotated using the MITOS web server (Bernt et al. 2013).

The complete mitogenome of *I. pacifica* (OM312055) is composed 16,561 bp of nucleotide. The genome consists of 13 PCGs, 22 tRNA genes, 2 rRNA genes, and 1 control region (D-loop). The gene order and arrangement were completely identical with the other cobitid species. The most common start codon in all PCGs is 'ATG', with the exception of COX1 that start with 'GTG'. For termination codon, seven PCGs (*ND1, COX1, ATP8, ATP6, ND4L, ND5, ND6*) have a complete ‘TAA’. But five PCGs (*ND2, COX2, COX3, ND3, CYTB*) have ‘T—’, an incomplete terminal codon, and only ND4 had ‘TA–’. The overall nucleotide compositions were 29.3% A, 27.5% T, 26.1% C, and 17.1% G, respectively.

The dataset for molecular phylogenetic analysis to understand the taxonomic relationship between *I. pacifica* (OM312055) and other sister species included 13 PCGs and 2 rRNA sequences of 16 other cobitid species, two families belonging to order Cypriniformes, and one species of Siluridae was added as an outgroup (Figure 1). For maximum likelihood analysis, the dataset was analyzed using MEGA version X (Kumar et al. 2018) with GTR + G + I model (Nei and Kumar 2000). The bootstrap resampling was accomplished with 1,000 iterations.

**Figure 1.**
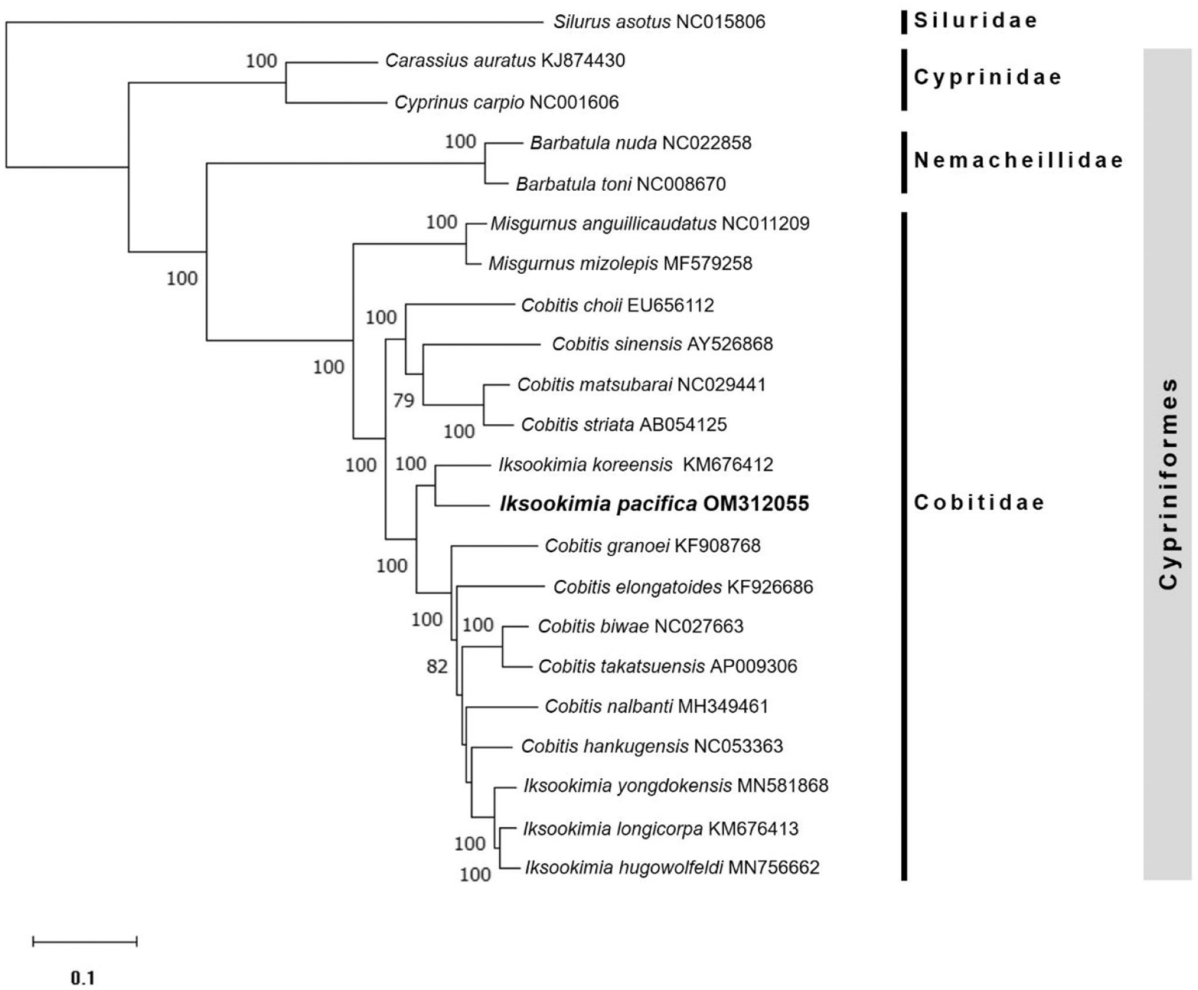
Phylogenetic tree of maximum likelihood (ML) method based on the nucleotide sequences of 13 PCGs and 2 rRNAs of 17 Cobitid species, including *I. pacifica* (OM312055), two families belonging to order Cypriniformes, and one Siluriformes species. Bootstrap support values based on 1,000 replicates are displayed on each node as >70.

In the phylogenetic analysis, *I. pacifica* was clearly distinguished from other species and was most closely related to *I. koreensis* (Figure 1). Our result suggests that *I. pacifica* and *I. koreensis* seem to have differentiated from the same recent common ancestor, and it was supported by the research that their habitats were interconnected before the geographical uplift event that occurred on the Korean Peninsula during the Miocene (Kwan et al. 2018). And it was also confirmed that Cobitidae species form a distinct monophyletic clade within the order Cypriniformes. On the other hand, *I. pacifica* and *I. koreensis* did not form a group with other Iksookimia species, consistent with other studies (Park et al. 2018; Kim et al. 2020). Therefore, a taxonomic review of these discrepancies is necessary, and our results are expected to provide useful information for reconstructing the phylogenetic relationship between Cobitidae species in future studies.

## Data Availability

The genome sequence data that support the findings of this study are openly available in GenBank of NCBI at (https://www.ncbi.nlm.nih.gov/) under the accession number OM312055. The associated BioProject, SRA, and Bio-Sample numbers are PRJNA798219, SRR 17652036, and SAMN 25050457, respectively.
